# Virome of high-altitude canine digestive tract and genetic characterization of novel viruses potentially threatening human health

**DOI:** 10.1128/msphere.00345-23

**Published:** 2023-09-19

**Authors:** Xiaojie Jiang, Jia Liu, Yuan Xi, Qing Zhang, Yongshun Wang, Min Zhao, Xiang Lu, Haisheng Wu, Tongling Shan, Bin Ni, Wen Zhang, Xiao Ma

**Affiliations:** 1 Department of Microbiology, School of Medicine, Jiangsu University, Zhenjiang, Jiangsu, China; 2 Qinghai Institute of Endemic Disease Prevention and Control, Xining, Qinghai, China; 3 Shanghai Veterinary Research Institute, Chinese Academy of Agricultural Sciences, Shanghai, China; Nanjing University of Chinese Medicine, Nanjing, Jiangsu, China

**Keywords:** canine, viruses, phylogenetic, viral metagenomics, hepatitis E virus

## Abstract

The majority of currently emerging infectious illnesses are zoonotic infections, which have caused serious public health and economic implications. The development of viral metagenomics has helped us to explore unknown viruses. We collected 1,970 canine feces from Yushu and Guoluo in the plateau region of China for this study to do a metagenomics analysis of the viral community of the canine digestive tract. Our analysis identified 203 novel viruses, classified into 11 known families and 2 unclassified groups. These viruses include the hepatitis E virus, first identified in dogs, and the astrovirus, coronavirus, polyomavirus, and others. The relationship between the newly identified canine viruses and known viruses was investigated through the use of phylogenetic analysis. Furthermore, we demonstrated the cross-species transmission of viruses and predicted new viruses that may cause diseases in both humans and animals, providing technical support for the prevention and control of diseases caused by environmental pollution viruses.

**IMPORTANCE:**

Most emerging infectious diseases are due to zoonotic disease agents. Because of their effects on the security of human or animal life, agriculture production, and food safety, zoonotic illnesses and livestock diseases are of worldwide significance. Because dogs are closely related to humans and domestic animals, they serve as one of the important links in the transmission of zoonotic and livestock diseases. Canines can contaminate the environment in which humans live such as water and soil through secretions, potentially altering the human gut microbiota or causing diseases. Our study enriched the viral community in the digestive tract microbiome of dogs and found types of viruses that threaten human health, providing technical support for the prevention and control of early warning of diseases caused by environmental contaminant viruses.

## INTRODUCTION

Using scientific models to estimate, the total count of viruses on Earth is around 10^31^, making them the most diverse biological entities on our planet (1, 2). To date, it is estimated that scientists have only scratched the surface of viral diversity, having explored less than 1% of the vast array of potential virus types, meaning that our current knowledge represents only a fraction of what may exist (3). Should a virus outbreak occur within human populations, the resulting harm may be grave, including fatalities among both humans and domesticated animals, presenting serious public health and safety concerns, as well as significant economic losses (4, 5). The human living environment plays a significant role in virus transmission, as evidenced by the pathogen-initiated cases of COVID-19, highly pathogenic avian influenza, etc. The environments inhabited by humans and animals are intrinsically interconnected, facilitating the reciprocal transmission of pathogens that can occur through contaminated environments such as fecal-contaminated water sources, and direct contact with humans, such as through bites. These transmission pathways contribute to the emergence of zoonotic diseases (6, 7). Zoonotic diseases and livestock illnesses have global significance due to their potential impacts on human and animal security, agricultural productivity, and food safety (8). It is worth noting that most emerging infectious diseases are caused by zoonotic pathogens (9). Therefore, investigating the viral communities present in human close-contact environments is crucial, since these environments, including air, water, and soil, harbor a significant number of identified and unidentified viral species that could potentially threaten human health (10 – 12).

Due to their close genetic relationship with humans and domestic animals, dogs play an important role in the transmission of zoonotic and livestock diseases (13). Canines are numerous, estimated to be around 700 million, and their widespread distribution, independent of human activity, enables the detection of canine activity in nearly all regions of the world (14). The mammalian gut microbiota, consisting of bacteria, archaea, fungi, protozoa, and viruses, has a microbial density that is 10 times greater than the number of host cells (15). The gut microbiome serves numerous functions, including but not limited to, influencing host growth and metabolism, and shielding the host from pathogen colonization (16). The gut microbiome is not constant throughout life and changes with factors such as age, environment, and lifestyle (17). With the ever-evolving society and human engagement in productive activities, recent studies have suggested that the human gut microbiota poses a risk of spillover to animals (18). It can be inferred that the environment exerts an influence on the composition of the gut microbiota. Canines have the potential to contaminate the surrounding environment, including water and soil, with their secretions, which can have consequential impacts on the human gut microbiota and contribute to the incidence of infectious diseases. Herders in high-altitude regions in China are at a heightened risk of contracting zoonotic diseases due to their frequent interaction with domestic animals such as dogs, unhygienic living conditions, and traditional lifestyle practices, thereby posing a significant public health and economic burden to local communities (19). Consequently, studying the presence of viruses in the gastrointestinal tract of canines may enable the prediction of zoonotic virus outbreaks in advance.

The complexity of the gut microbiota renders previous culture techniques insufficient for studying its intricate functions, thus necessitating the implementation of novel technological alternatives to elucidate its mechanisms (20). In recent years, advancements in technologies such as viral molecular biology and next-generation sequencing have led to increasing utilization of viral metagenomics as an efficient tool for discovering unknown viruses in humans (21). The application of viral metagenomic techniques in gut microbiota studies has proven to be an effective means of elucidating the structural and functional complexities of microbial communities, thus providing more comprehensive insights into their dynamics and interactions (22).

This research endeavor involved the collection of 1,970 fecal samples from highland canines to elucidate the composition of enteroviruses via the application of viral metagenomic techniques. Our analysis led to the discovery of novel canine viruses, which were subjected to phylogenetic analysis to determine their relationship with previously identified viruses. Furthermore, our findings enabled us to predict the likelihood of the emergence of potential novel viruses with the capacity to induce diseases in human and livestock populations.

## MATERIALS AND METHODS

### Canine sample collection

To conduct a viral metagenomic analysis, this study collected a total of 1,970 canine fecal samples between 2020 and 2021 from two Tibetan Autonomous Prefectures in Qinghai Province, China. Specifically, 950 samples were obtained from Yushu Tibetan Autonomous Prefecture, and 1,020 samples were obtained from Guoluo Tibetan Autonomous Prefecture. The collected samples are stored in the −80℃ refrigerator as soon as possible. The fecal samples were individually resuspended in 1 mL of Dulbecco’s phosphate buffered saline, and vortexed vigorously for 5 min. The supernatants were then collected after centrifugation (10 min, 15,000 g, 4°C). This study obtained approval from the Ethics Committee of Jiangsu University and Qinghai Institute for Endemic Disease Prevention and Control for sample collection and all experimental procedures. Additionally, all sample pre-treatment was conducted in the secondary laboratory of biosafety at Qinghai Institute of Local Disease Control and Prevention.

### Sample preparation and library construction

The 1,970 individual samples were combined into 197 pools, with each pool containing an average of 10 samples. To remove eukaryotic and bacterial cell-sized particles, sample pools were centrifuged (20 min, 12,000 g, 4°C), and then the supernatant was washed with a 0.45-µM filter filtration (23, 24). Then digest filtrates with DNase and RNase (Turbo DNase, Thermo Fisher Scientific, MA, USA; BaselineZeroDNase, Epicentre, WI, USA; Benzonase Nuclease, Novagen, MA, USA; and RNase A, Thermo Fisher Scientific) at 37℃ for 60 min (25 – 27). According to the manufacturer’s proposal, all remaining nucleic acids (DNA and RNA) were separated using QIAamp Viral RNA Mini Kit (Qiagen). Since the nucleic acid is composed of RNA, it needs to be reversely transcribed into cDNA through a reverse transcriptase kit (SuperScript IV reverse transcriptase, which contains six random primers). Then double-stranded DNA (dsDNA) was synthesized from different virus templates for DNA library construction. The second cDNA strand (dsDNA) was synthesized by adding Klenow fragment polymerase (New England Biological Laboratory). At the same time, for single-stranded DNA (ssDNA) viruses, ssDNA is converted into dsDNA during this Klenow reaction. The Nextera XT DNA Sample Preparation Kit from Illumina was used to generate 197 pools of dsDNA products. These pools were then sequenced on the Illumina NovaSeq 6000 platform using 250-bp paired-end sequencing with dual barcoding for each individual sample pool (28).

### Bioinformatics analysis

To facilitate bioinformatics analysis, we decoded 250-bp paired-end reads from a total of 197 libraries constructed using software provided by Illumina vendor. The data underwent processing through an internal analysis pipeline running on a 32-node Linux cluster. Eukaryotic and prokaryotic genome sequences were filtered out using Bowtie 2 v2.3.4.1, utilizing viral sequences from GenBank as a reference. Low-quality tails at both ends of the sequences were subsequently trimmed using Phred v1.0.0, with a quality score threshold of 10. Duplicate sequences were identified by comparing bases 5–55, and only one randomly selected duplicate was retained. The VecScree (https://www.ncbi.nlm.nih.gov/tools/vecscreen/) website was employed with default parameters to remove joint sequences at both ends. The resulting trimmed and clean reads were then assembled and spliced using EnsembleAssembler v1.0.0. The contigs and singlet reads obtained from each library were compared to the NCBI’s viral proteome database using DIAMOND v0.9.24 for BLASTx analysis with an *E*-value threshold of less than 10^−5^ (29). The candidate viral sequences were further compared to the non-virus non-redundant protein database to eliminate false-positive viral sequences (30). For negative contigs and singlet reads, research was performed in the vFam database using HMMER v3.1b2 to identify false-negative viral sequences (31, 32). Default parameters were used for all searches. Finally, the annotation information for virus-related contigs and singlet reads was presented using Megan v6.21.16.

### Virus genome determination and PCR validation

According to Megan v6.21.16, the reads obtained previously are classified into corresponding sections. Then, *de novo* assembly and reference mapping were performed in Geneious prime v2019.0, and the reads were assembled to obtain the complete or partial virus genome. When there was a gap between the contigs of the viral genome, nested PCR was performed. Prepare the appropriate PCR system according to the regulations and then add the pre-mixed enzyme rTaq (Takara Biomedical Technology) into the PCR reaction system. The PCR conditions used were as follows: 95°C for 5 min, 35 cycles for 95°C for 30 s, 50°C for 30 s, 72°C for 45 s, and a final extension at 72°C for 10 min. All PCR steps have negative control and are sequenced by the Sanger method. Meanwhile, Geneious prime v2019.0 enables the prediction and annotation of open reading frames (ORFs) and can also design primers. The predicted ORF should be retrieved and compared through BLASTx. To address gaps in the 3′ terminal region, an expression vector was constructed and transfected into DH5α active cells. The transfected cells were then screened to identify positive clones using bacterial liquid PCR and sequencing methods.

### Analysis of viral communities

The statistical analysis related to the experiment was carried out by Megan v6.21.16 and R v4.2.1. The composition analysis of 197 libraries was standardized and compared by Megan (33). The viral community structure and richness findings were illustrated using R v4.2.1 by employing the heatmap and vegan packages. Additionally, the differences in viral communities were visualized using the ggplot2 package. When *P* < 0.05, the study has statistical significance.

### Phylogenetic analysis

The protein sequence of the virus identified in this study was utilized in a phylogenetic analysis along with its closest match based on BLASTx results in the NCBI GenBank database, as well as representative protein sequences of viruses belonging to the same family as the virus under investigation. To align the protein sequences of interest, we employed the MUSCLE algorithm implemented in MEGA v10.1.8 with the default settings (34). Then the sorted protein sequences are used to construct a Bayesian inference tree through MrBayes v3.2.7 (35). In MrBayes, we asked amino acid sequences to construct phylogenetic analysis under the set program (“prset aamodelpr = mixed”), which allows 10 built-in amino acid models. The number of generations can be increased to 10 million at most, and the standard deviation of the final division frequency is less than 0.01 before the operation stops (36). To visually represent the phylogenetic tree, it was visualized and edited by Figtree v1.4.4 (http://tree.bio.ed.ac.uk/software/figtree/) and Adobe Illustrator 2020 v26.0.1.

### Quality control

In order to eliminate the possibility of nucleic acid contamination in the laboratory, sterile ddH_2_O (Sangon Biotech) was prepared by high-pressure sterilization and further processed under the same conditions as the blank control group. In the whole process of the experiment, common laboratory preventive measures were followed to prevent cross-contamination and nucleic acid degradation. DNase and RNase are not present in each substance in direct contact with nucleic acid samples. RNase inhibitors and water-DEPC-treated (Sangon Biotech) are used to dissolve nucleic acid samples. No viral contamination was detected by analyzing the sequencing results of the negative control groups. This indicates that the study was effective in preventing potential viral contamination. Negative control group raw data were submitted to the database.

## RESULTS

### Overview of the canine virome

**Fig 1:**
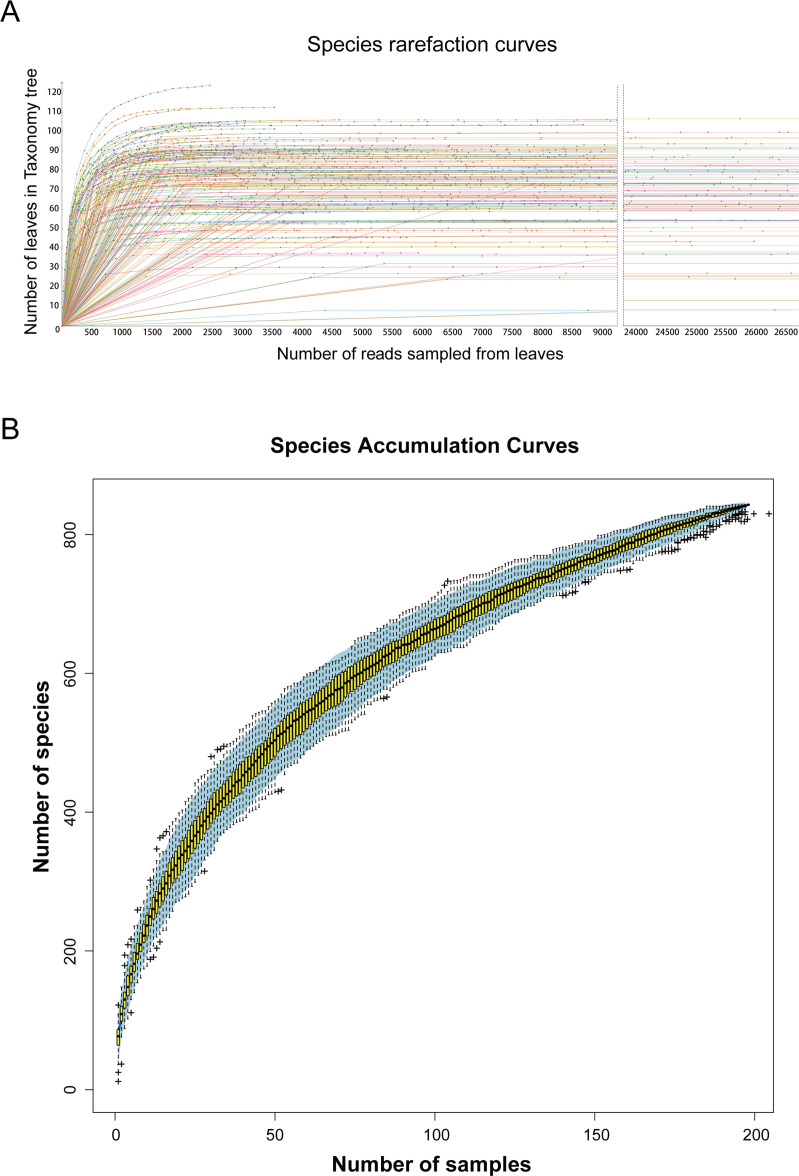
The diversity of viral species in the 197 libraries. (A) The resulting species rarefaction curves were plotted after log-scale transformation of the raw data in Megan v6.21.16 software. (B) Accumulation curve of viral species in canine metagenomes. Individual box plots correspond to the richness values of the samples, with light blue areas representing 95% confidence intervals.

To gain insight into the enterovirus community present in high-altitude canines, we procured a total of 1,970 canine fecal samples from Yushu and Guoluo prefectures in Qinghai Province. Among these, 950 canine fecal samples were collected from Yushu, while 1,020 canine fecal samples were procured from Guoluo. From these samples, 197 libraries were constructed and sequenced through metagenomic sequencing utilizing the Illumina NovaSeq platform, resulting in a total of 416,769,906 raw reads with an average GC% of 57.9%. The assembled metagenomes were screened and compared against the GenBank non-redundant protein database using BLASTx (with an *E* value of less than 10^−5^), leading to the identification of 17,127,024 virus reads (accounting for 4.109% of the total). Species richness was determined by analyzing species rarefaction and accumulation curves, which revealed that the observed viral species in the majority of 197 libraries had leveled off. Thus, we conclude that the current sequencing depth has covered the entirety of the virus species present in the collected samples. Even with additional sequencing data, the diversity of viral species cannot be further increased (Fig. 1A). As the number of samples increased, the species accumulation curves gradually smoothed, indicating that the sample size collected in this study was adequate and representative of the study as a whole (Fig. 1B). And the accumulation curves showed more than 800 different viruses in 197 libraries.

The analysis identified 11 different virus families and 2 unclassified groups for a total of 203 viral genome sequences, including 158 DNA viruses and 35 RNA viruses known to infect and potentially vertebrate species—*Anelloviridae* (*n* = 13), *Circoviridae* (*n* = 19), *Genomoviridae* (*n* = 46), *Parvoviridae* (*n* = 45), *Smacoviridae* (*n* = 8), unclassified circular Rep-encoding single-stranded DNA (CRESS-DNA) viruses (*n* = 19), *Vilyaviridae* (*n* = 7), *Polyomaviridae* (*n* = 1), *Coronaviridae* (*n* = 1), *Astroviridae* (*n* = 18), *Hepeviridae* (*n* = 1), and *Picornaviridae* (*n* = 14)—while the remaining 11 genomes were unclassified viruses (*n* = 11).

### Diversity analysis of virus communities

**Fig 2:**
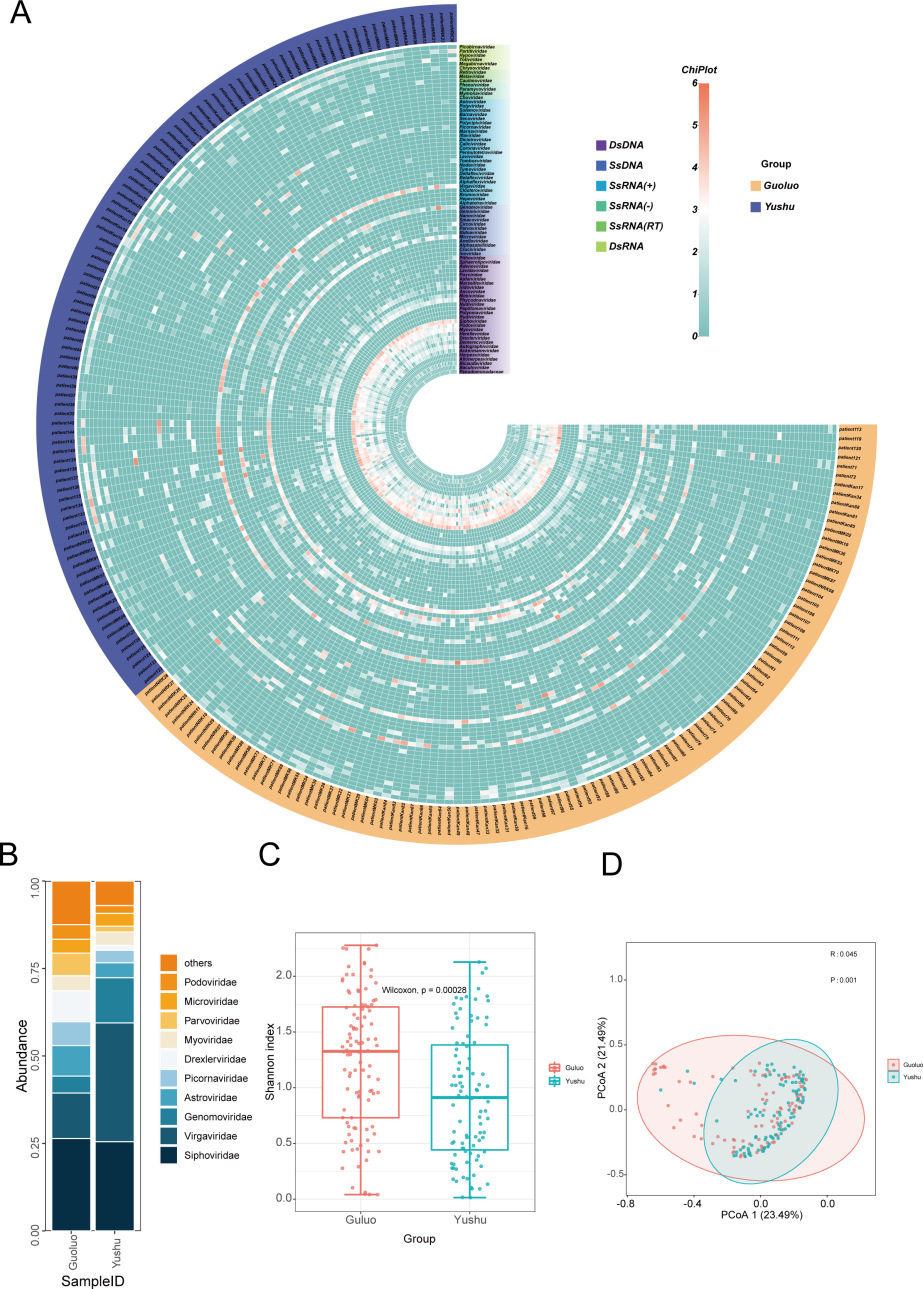
Statistical analysis of enterovirus communities. (A) Heatmap was constructed after transforming read counts per virus family in individual libraries on a log10 scale. Nucleic acid types, virus families, and sampling region groupings are annotated with different colors (see color legend). (B) Bar chart by virus database by geography showing the relative proportion and taxonomy based on viral families. (C) Viruses to be compared were normalized using Megan prior to the comparison of virus alpha diversity, and the virus abundance (family level) of the Guoluo Group and Yushu group, which were divided into different sampling regions, was measured using the Shannon index. The *P* value was calculated using the Wilcoxon test. (D) Viruses to be compared were normalized using Megan prior to the comparison of virus beta diversity, principal co-ordinates analysis (PCoA) analysis of Guoluo Group and Yushu Group at the family level. R was more than 0, which indicated that there were differences between the groups. Studies were considered statistically significant when the *P* value was less than 0.05.

A heatmap was constructed to investigate the dissimilarities in the viral composition of individual libraries. This heatmap was based on the viral family level, sampling region, and nucleic acid type of 197 libraries’ viral genome sequences. The resulting data, which were log-transformed, showed that 78 virus families, including 28 dsDNA virus families, 12 ssDNA virus families, 25 ssRNA (+) virus families, 4 ssRNA (−) virus families, 3 ssRNA (RT) virus families, and 6 dsRNA virus families were present in the 197 libraries (Fig. 2A). Of these libraries, 102 were from Guoluo and 95 were from Yushu. The largest amount of *Siphoviridae*virus reads was 1,509,845 (26.41%) in the Guoluo group, while the largest amount of viral reads was 2,019,571 (33.94%) in the Yushu group, and the number of viral reads of *Virgaviridae* was only 742,161 (12.98%) in the Guoluo group, demonstrating a clear difference in canine enterovirus composition between the two regions. The amount of *Siphoviridae* virus reads in the Yushu group was 1,517,068 (25.50%), indicating that the same segments existed in the canine enterovirus composition in the two regions. *Genomoviridae* had 767,762 reads (12.90%) in the Yushu group but only 277,230 reads (4.85%) in the Guoluo group. There were obvious differences in the amount of virus reads among the same*Siphoviridae*, *Picornaviridae*, *Parvoviridae*, and similar amounts between *Myoviridae* and *Microviridae*. The relative abundance plots demonstrated that the small fractions of canine digestive tract virus composition were similar in the two regions of Guoluo and Yushu. However, the differences were obvious (Fig. 2B). To further investigate the differences in the composition of the viral communities from the canine digestive tract between the two regions, alpha diversity analysis and beta diversity analysis were performed on the viral communities from the two regions. In the alpha diversity analysis, a significant difference in the viral community was observed between the Guoluo group and the Yushu group, as the *P* value was far from 0.05 (Fig. 2C). In beta diversity analysis, the *r* value greater than 0 indicated that the distance within the group was less than the distance between the groups, suggesting a significant difference in grouping, which was supported by the *P* value (0.001) less than 0.05 (Fig. 2D). These results indicate that the differences in viral composition between the two regions are statistically significant.

### Canine *Astroviridae*

**Fig 3:**
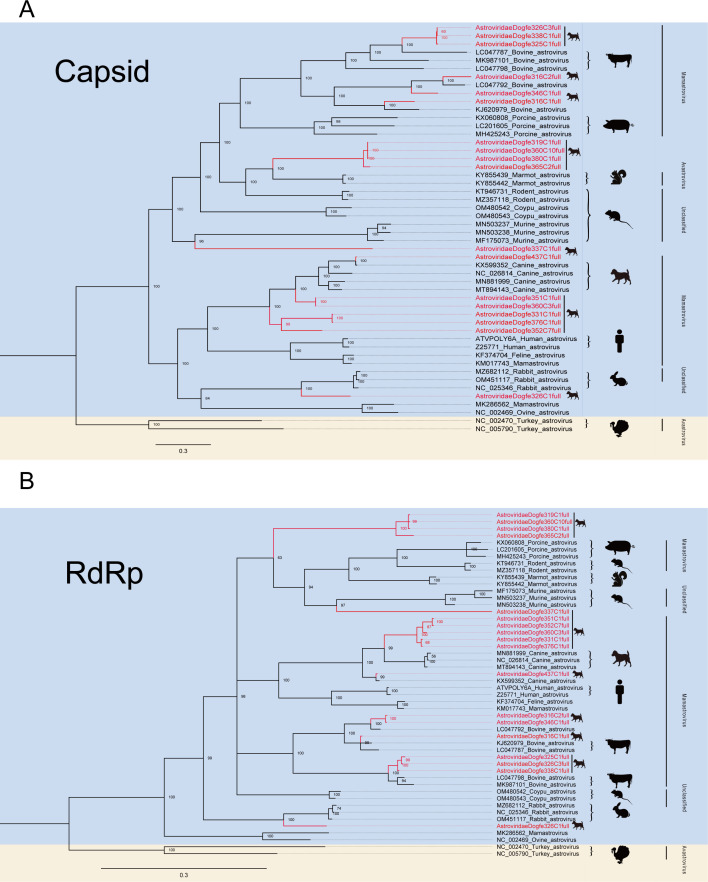
Phylogenetic relationship of *Astroviridae*. (A) Bayesian inference tree based on amino acid sequences of capsid of viruses belonging to *Astroviridae*. (B) Phylogenetic tree based on RdRp protein. The Bayesian inference tree based on amino acid sequences of RdRp of viruses belonging to *Astroviridae*. Red represents sequences from this study. The scale bar represents the length of the unit representing the value of the difference between organisms or sequences, equivalent to the scale of an evolutionary tree.

Astroviruses are single-stranded positive RNAs, and the genomes are divided into three overlapping ORFs (37, 38). Astroviruses can infect a wide range of mammals and birds (39). In this investigation, the 752,794 reads obtained from sequencing were identified and reassembled, resulting in the acquisition of 18 nearly complete astrovirus genomes through alignment with the NCBI GenBank database using BLASTx data. In order to be able to analyze the relationship between the newly discovered genome and other known astroviruses, the respective phylogenetic analysis trees were constructed based on the capsid protein (Fig. 3A) and RdRp protein sequences (Fig. 3B), respectively. Overall, although the capsid protein-based phylogenetic analysis tree shares a similar topology to the RdRp protein tree (e.g., Astroviridaedogfe316c2full, Astroviridaedogfe326c1full, AstroviridaeDogfe337C1full), certain strains do not occupy the same positions in the tree based on capsid proteins and RdRp (e.g., AstroviridaeDogfe316C1full, AstroviridaeDogfe319C1full, AstroviridaeDogfe360C10full), which can be attributed to the genomic reorganization performed. From the phylogenetic analysis trees (Fig. 3A and B), it can be seen that there are likely evolutionary relationships between the astrovirus genomes of new-onset dogs (e.g., AstroviridaeDogfe326C1full, AstroviridaeDogfe326C3full) and those present *in vivo* in various mammals (e.g., murine, bovine, swine). And part of the canine astrovirus genomes (e.g., AstroviridaeDogfe352C7full, AstroviridaeDogfe376C1full) and part of the human astrovirus genomes belong to the same genus and may have shared evolutionary ancestry. These findings suggest that astroviruses have a broad range of transmission among mammalian species, highlighting the potential risk of canine astroviruses for human infection. The family *Astroviridae* at the International Committee on Taxonomy of Viruses (ICTV) determined that viruses should be considered as members of the same species if the genetic distance between the host and the amino acid sequence of the capsid protein (*p* distance) is >75% identity (40). The four astroviruses, AstroviridaeDogfe319C1full, AstroviridaeDogfe360C10full, AstroviridaeDogfe365C2full, and AstroviridaeDogfe380C1full, clustered together and shared less than 50% identity, indicating that they may belong to a new genus. AstroviridaeDogfe337c1full, on the other hand, clustered separately and shared less than 50% identity, suggesting that it may be a new species.

### Canine *Coronaviridae*

**Fig 4:**
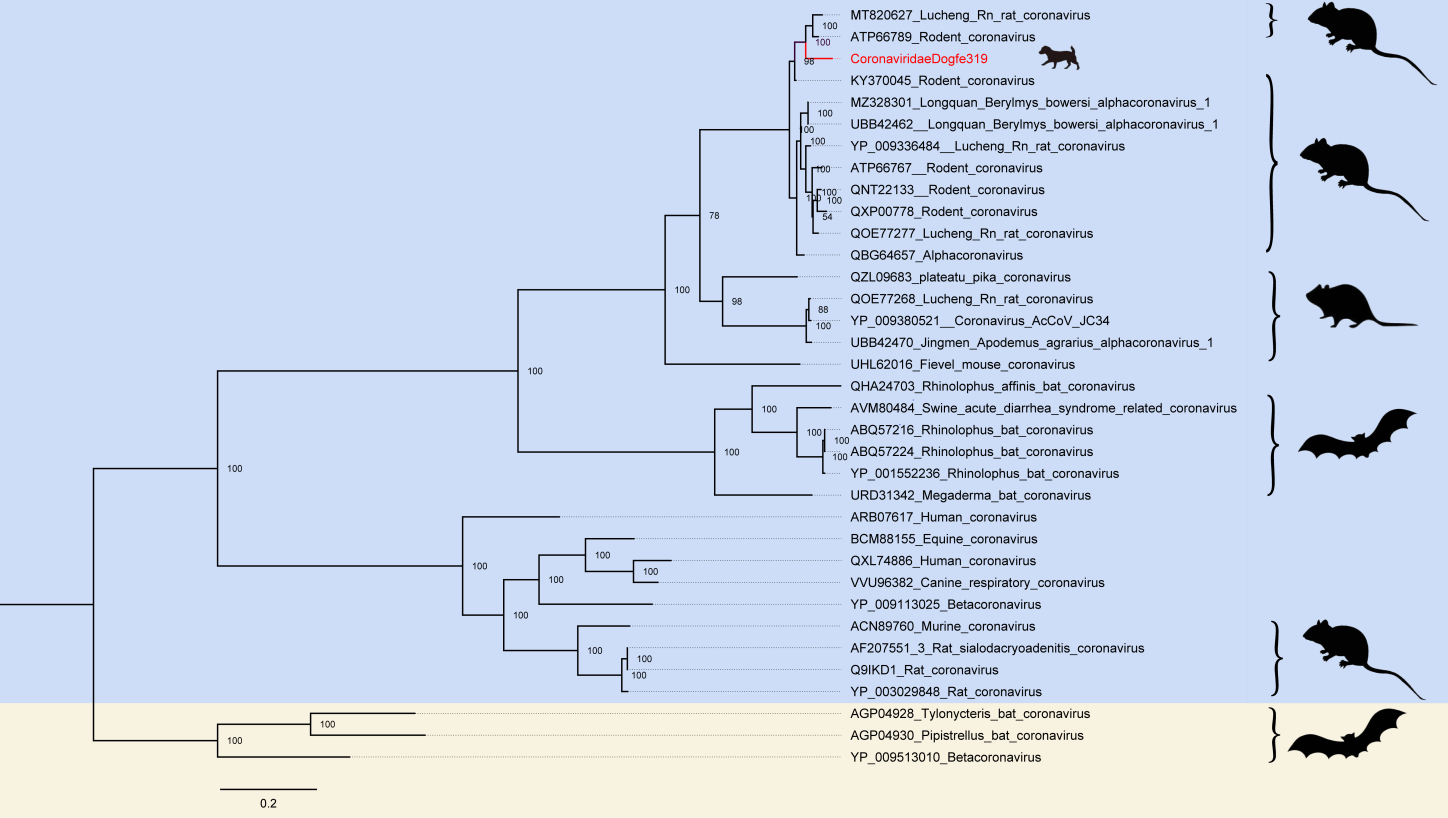
Phylogenetic relationship of *Coronaviridae*. A Bayesian inference tree was constructed based on the amino acid sequences of the coronavirus spike protein. Red represents sequences from this study. The scale bar represents the length of the unit representing the value of the difference between organisms or sequences, equivalent to the scale of an evolutionary tree.

Coronaviruses are single-stranded RNA genomes with a size of about 26–32 kb (41). Coronavirus was discovered in post-1960 to be transmissible and cause disease in animals and humans, with three large outbreaks in human society, respectively, severe acute respiratory syndrome in 2002, Middle East respiratory syndrome in 2012, and coronavirus disease (COVID-19) in 2019 (42 – 44). Three large-scale outbreaks, all of which were coronavirus transmission to humans via animals, raised serious public safety concerns and demonstrated its robust cross-species transmission and ability to adapt to human hosts, so that surveillance and research on animals harboring coronaviruses such as bats are warranted (45). The 916 reads obtained from sequencing were identified and assembled, and subsequently aligned with the NCBI GenBank database using BLASTx data. This process yielded one nearly complete coronavirus genome. To enable analysis of the difference between the newly discovered genome and other known coronaviruses, a phylogenetic analysis tree was prepared to be constructed. Since coronaviruses are enveloped viruses, the spike protein catalyzes and induces neutralizing antibody responses, the key first step in the virus-infected host, and therefore, is an important target for diagnosis, therapy, and vaccine development. A phylogenetic analysis of the coronavirus tree is based on the spike protein sequence (46). CoronaviridaeDogfe319 is 92.29% identical at the amino acid level to the coronavirus of *Eothenomys miletus* (GenBank no. MT820627) collected in China in 2020, which are considered to have a common ancestor and similar in structure and function. Phylogenetic analysis tree diagrams illustrate that the canine-derived coronaviruses share a common ancestor with the partly murine-derived coronaviruses, which are more distant from those of bat origin (Fig. 4).

### Canine *Hepeviridae*

**Fig 5:**
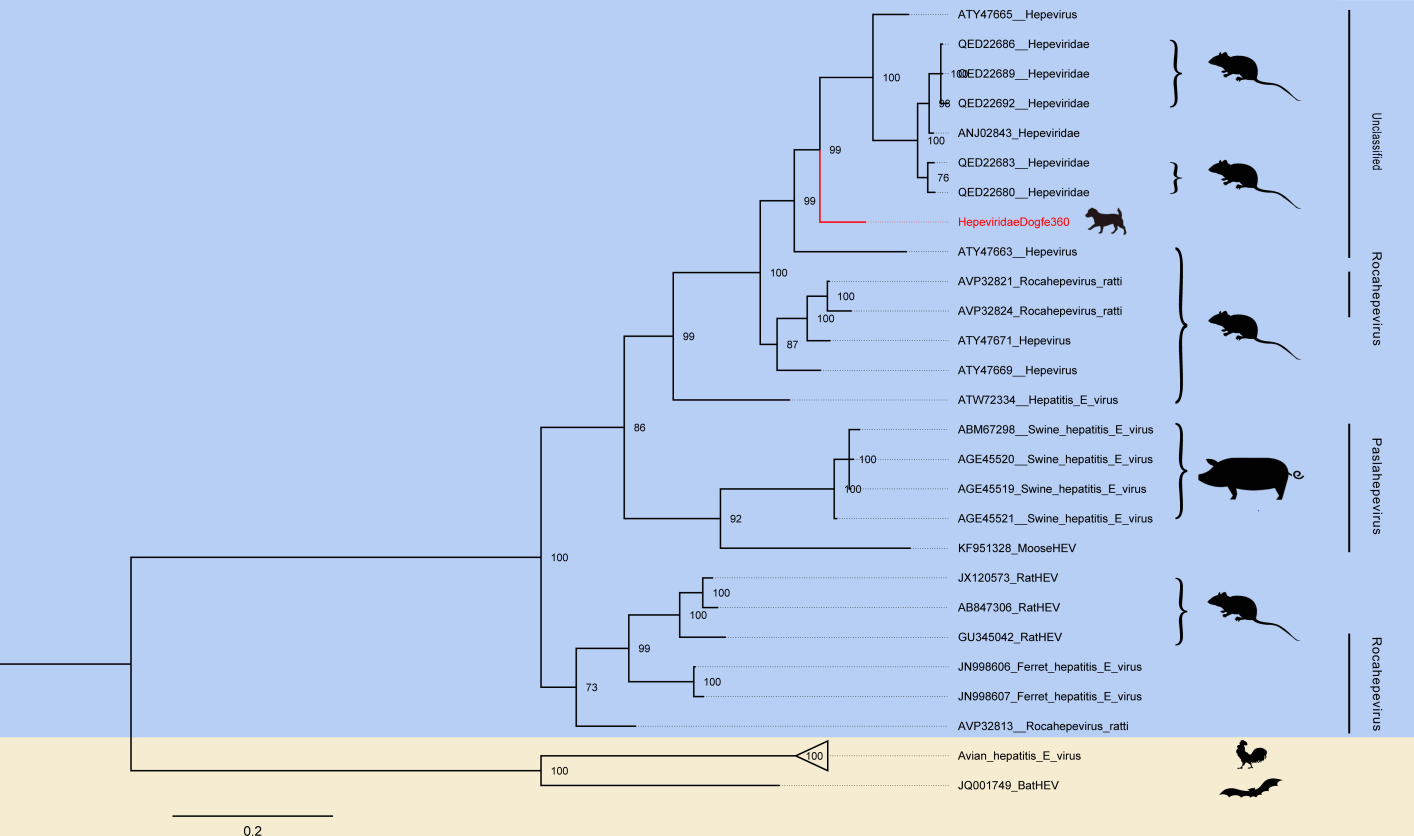
Phylogenetic relationship of *Hepeviridae*. A Bayesian inference tree was constructed based on the amino acid sequences of the RdRp of *Hepeviridae*. Red represents sequences from this study. The scale bar represents the length of the unit representing the value of the difference between organisms or sequences, equivalent to the scale of an evolutionary tree.

Hepatitis E viruses (HEVs) are single-stranded RNAs with a molecular weight of approximately 7.2 kb and have three ORFs (47). Hepatitis E viruses infect a variety of animals (e.g., pigs, sheep, bats, and birds) and humans (48). Hepatitis E viruses can spread through four different methods: vertical transmission, zoonotic transmission, transfusion transmission, and waterborne transmission. Previous studies have shown that dogs have not been infected with hepatitis E virus. Remarkably, our study is the first to demonstrate that dogs can be infected with hepatitis E virus. The 299 reads obtained from the research identification and recombination sequencing were then compared with the NCBI GenBank database using BLASTx data to obtain a roughly complete hepatitis E virus. In order to analyze the relationship between the newly discovered genome and other known hepatitis E viruses, an amino acid phylogenetic analysis tree based on RdRp was constructed. HepeviridaeDogfe360 has 85.40% identity at the RdRp amino acid level with hepatitis E virus (GenBank no. ATY47671) collected in 2017 in rodents in China. According to the phylogenetic tree, the novel virus identified by our study is the closest relative to the hepatitis E virus kindred of murine origin, and within the genus, it can only be classified as unclassified within the *Hepeviridae* (Fig. 5).

### Canine *Polyomaviridae*

**Fig 6:**
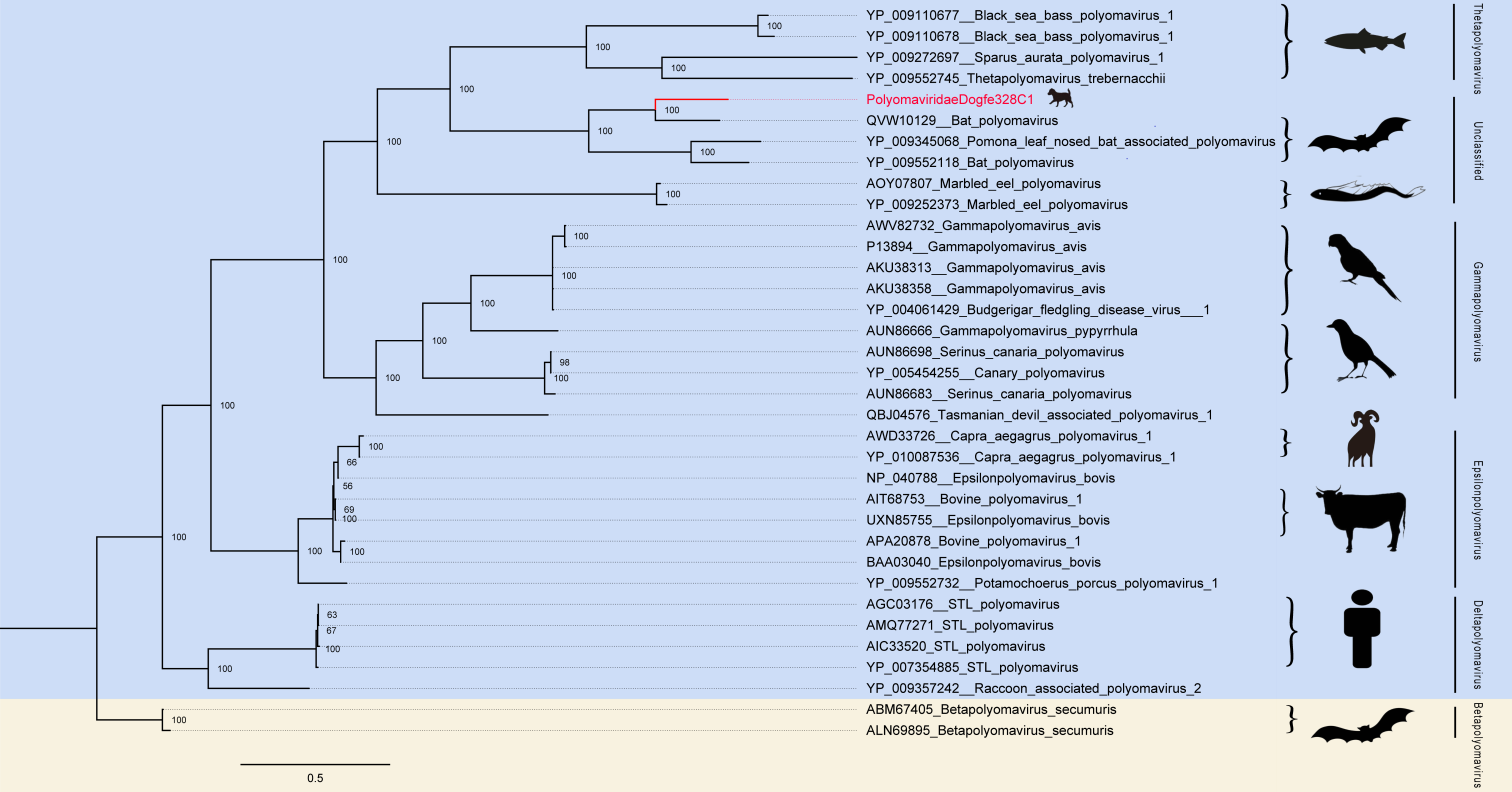
Phylogenetic relationship of *Polyomaviridae*. Amino acid sequences structure phylogenetic analysis tree based on LT-Ag of *Polyomaviridae*. Red represents sequences from this study. The scale bar represents the length of the unit representing the value of the difference between organisms or sequences, equivalent to the scale of an evolutionary tree.

Since the first discovery of polyomavirus in mice in 1953, multiple polyomaviruses have now been discovered that can infect both mammals and humans (e.g., rabbit polyomavirus, BK virus) (49, 50). Polyomaviruses are circular double-stranded deoxyribonucleic acids, approximately 5 kb in length, nonenveloped viruses that do not cause disease in immunocompetent healthy individuals but are severe in markedly immunocompromised patients, potentially leading to cancer (51). In December 2015, the ICTV *Polyomaviridae* study group, in order to update the classification of the *Polyomaviridae*, established a new standard for defining and creating polyomavirus species based on the observed distance between large T antigen (LT-Ag) coding sequences (52). The newly discovered approximately complete polyomavirus and other known polyomaviruses were combined to construct a phylogenetic analysis tree based on the amino acid sequences of LT-Ag. The best match at the amino acid level of LT-Ag by PolyomaviridaeDogfe328C1 was the bat polyomavirus (GenBank no. MZ218055) (https://www.ncbi.nlm.nih.gov/nuccore/MZ218055) identity of 68.36%. And as can be seen from the tree, Polyomaviridaedogfe328C1 also belongs to the unclassified order and is closest in distance to polyomaviruses of partial bat origin, both of which probably shared a common evolutionary ancestor (Fig. 6).

### Canine *Anelloviridae*

**Fig 7:**
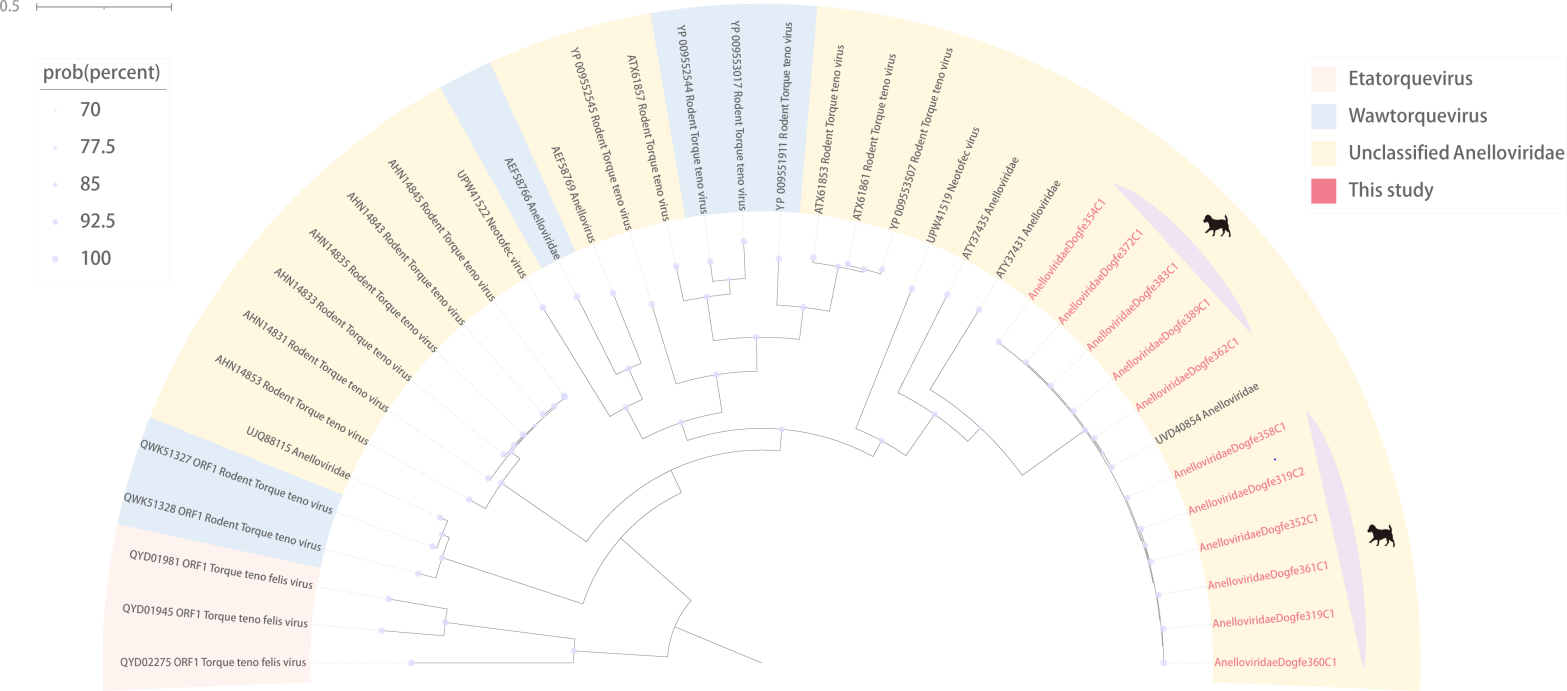
Phylogenetic relationship of *Anelloviridae*. Phylogenetic analysis tree based on ORF1 amino acid sequences structure of *Anelloviridae*. Red represents sequences from this study. The scale bar represents the length of the unit representing the value of the difference between organisms or sequences, equivalent to the scale of an evolutionary tree. Each color represents one genus in the legend. Purple represents the virus of canine origin.

Anelloviruses are single-stranded DNA viruses, whose genome size varies from 1.6 to 3.9 kb with one long and two or three shorter ORFs (53, 54). Studies have found a large number of diverse anelloviruses, through which phylogenetic trees can be constructed with the complete open reading frame 1 (55). Anellovirus is highly prevalent and widely distributed in humans, where it has been found through numerous studies to have complex interactions with the human immune system, but there is currently no evidence for its direct involvement in any pathogenic process, so it belongs to the group of viruses that are potentially hazardous to human health (56). We identified and assembled 8,524 reads obtained from sequencing, obtained the genomes of 11 nearly complete anelloviruses, and then constructed a phylogenetic tree based on the amino acid sequence of ORF1. By BLSATx comparison, we found that the genomes of these 11 anelloviruses (e.g., AnelloviridaeDogfe389C1) were all more than 99% similarity to anellovirus of murine origin (GenBank no. UVD40854), considering that there might be a possibility of cross-species transmission. Meanwhile, from the tree, it is evident that the anelloviruses found in our study belong to unclassified genera and may share a common evolutionary ancestor with anellovirus of murine origin (Fig. 7).

### Canine *Picornaviridae*

**Fig 8:**
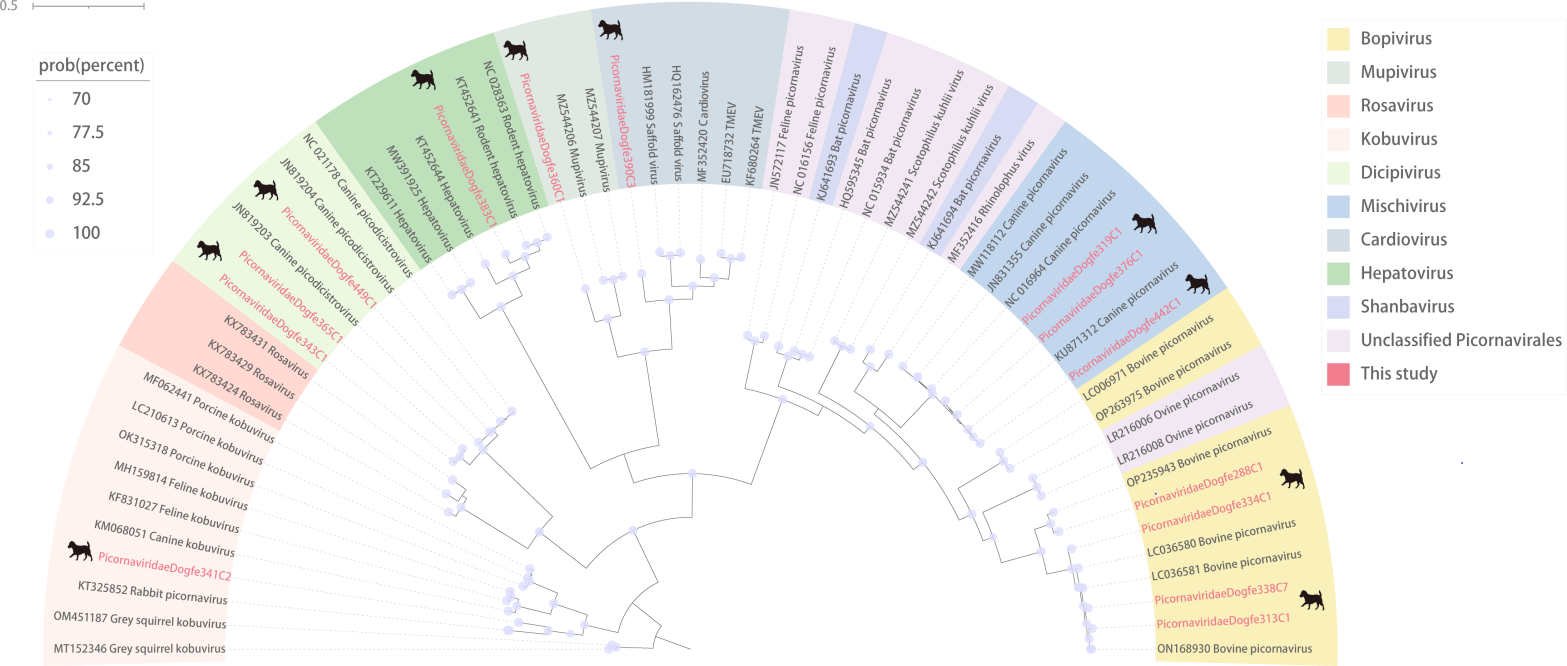
Phylogenetic relationship of *Picornaviridae*. The phylogenetic analysis tree was constructed according to the structure of the RdRp amino acid sequences of *Picornaviridae*. Red represents the sequence of this study. The scale bar represents the length of the unit representing the value of the difference between organisms or sequences, equivalent to the scale of an evolutionary tree. Each color in the legend represents a genus.

Picornaviruses are RNA viruses ranging from 6.7 to 10.1 kb in length (57). While infection with picornaviruses mostly does not cause disease and only a small subset causes severe disease of the central nervous system and the heart, the diseases caused by infection are not in the small number due to their ubiquitous presence and wide distribution among vertebrates (58). We characterized the 601,432 *de novo* reads obtained from sequencing and finally obtained a genome of 14 nearly complete picornaviruses, and then constructed a phylogenetic analysis based on the amino acid sequences of the RdRp. It is evident from the tree that picornaviruses are widely distributed among various organisms and that the newly identified picornaviruses are not clustered together in the tree diagrams, being distributed among the various genera of the *Picornaviridae* (Fig. 8). By BLSATx comparison, the genes of the RdRp of the 14 picornaviruses showed greater than 90% similarity, except for Picornaviridaedogfe341C2, Picornaviridaedogfe360C1, and Picornaviridaedogfe390C3, and among them. Picornaviridaedogfe442C1 was 99.33% identical to a canine-associated picornavirus (GenBank no. KU871312), while Picornaviridaedogfe449C1 was 99.11% identical to a canine-associated picornavirus (GenBank no. JN819204), both of which were also collected in Hong Kong, China, in 2008. Picornaviridaedogfe341C2 is 74.73% identical to picornavirus of rabbit (GenBank no. KT325852) collected in Hungary. Picornaviridaedogfe360C1 was 73.09% identical to picornavirus of murine (GenBank no. MZ544208) collected in Vietnam. Picornaviridaedogfe390C3 has 75.00% identity with picornavirus of murine (GenBank no. MH976711) collected in China. Both Picornaviridaedogfe334C1 and Picornaviridaedogfe338C7 are more than 98% identical to picornaviruses of bovines. This demonstrates that picornaviruses are widespread in the world and occur from plateaus to plains, with potential cross-regional and cross-species transmission.

### Canine *Parvoviridae*

**Fig 9:**
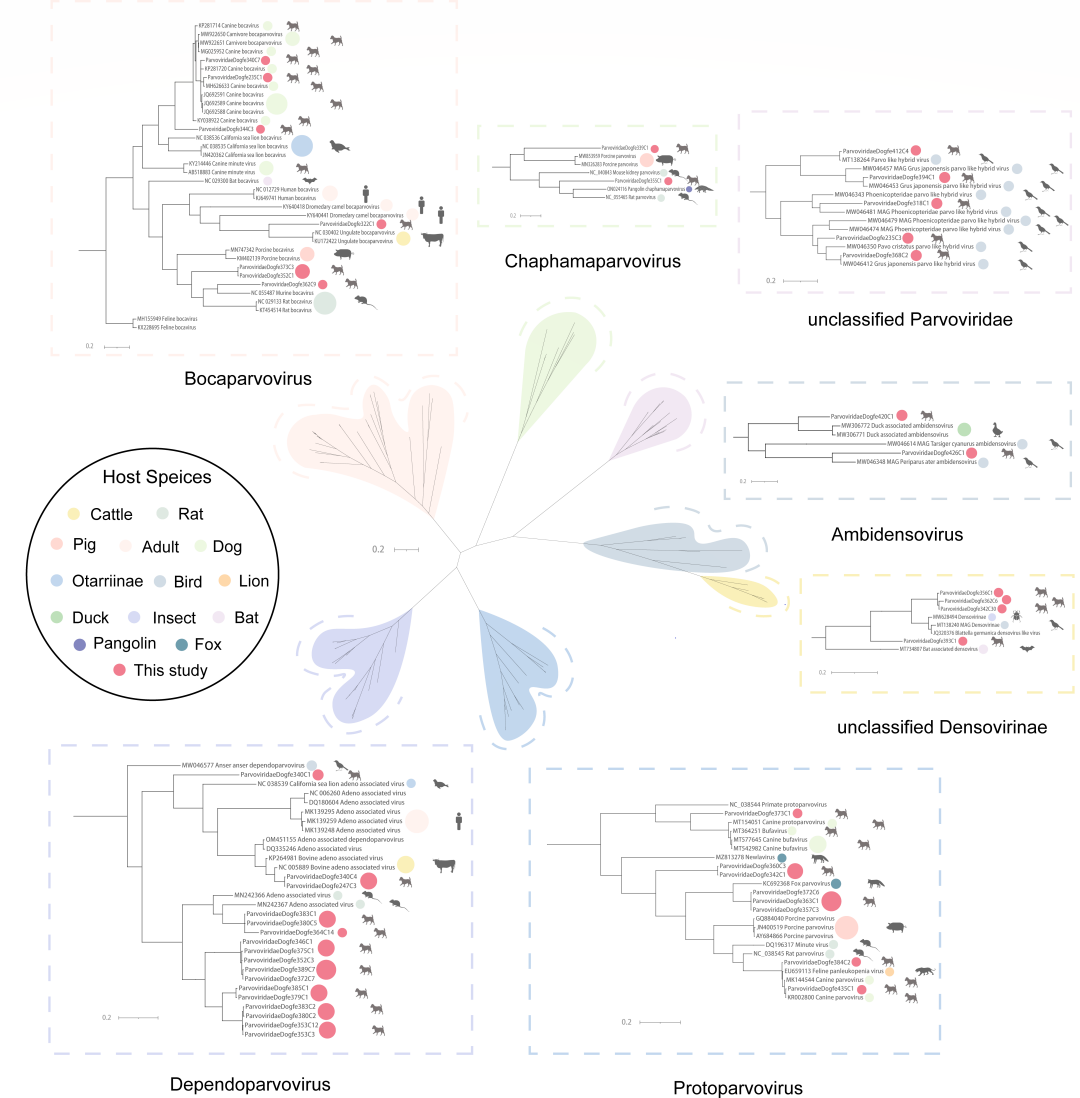
Phylogenetic relationship of *Parvoviridae*. A phylogenetic analysis tree was constructed based on the non-structural protein 1 (NS1) amino acid sequence structure of *Parvoviridae*. Each of the different colored dots in the legend represents a host organism, and the red dots represent this study. The scale bar represents the length of the unit representing the value of the difference between organisms or sequences, equivalent to the scale of an evolutionary tree.

Parvoviruses are DNA viruses, approximately 4.5–5.5 kb in length, with three or four ORFs (59). The three main subfamilies of the *Parvoviridae* are the *Parvovirinae*, which infect vertebrates, the *Densovirinae*, which infect arthropods, and the *Hamaparvovirinae*, which infect both invertebrates and vertebrates (60, 61). *Parvovirinae* is classified into eight genera as follows: *Erythroparvovirus*, *Dependoparvovirus*, *Protoparvovirus*, *Bocaparvovirus*, *Tetraparvovirus*, *Aveparvovirus*, *Copiparvovirus*, and *Amdoparvovirus*; the first five genera can infect humans (62). We characterized and assembled 467,460 reads obtained from sequencing and ultimately obtained 45 nearly complete parvoviruses. To clearly identify the genera in which the emerging viruses are found, a clear phylogenetic analysis tree was constructed by building a phylogenetic tree based on the amino acid sequence of nonstructural protein 1 (NS1). The phylogenetic tree was divided into seven genera, three of which belong to *Parvovirinae*—*Bocaparvovirus* (*n* = 7), *Dependoparvovirus* (*n* = 17), *Protoparvovirus* (*n = 8*), *Chaphamaparvovirus* (*n* = 2), unclassified *Parvoviridae* (*n = 5*), *Ambidensovirus* (*n* = 2), and unclassified *Densovirinae* (*n* = 4) (Fig. 9). NS1 sequences of 32 canine *Parvovirinae* show homology between mammalian and avian sequences at the amino acid level. Parvoviridaedogfe384C2 is 99.55% identical to the feline panleukopenia virus of a lion (GenBank no. EU659113) collected in the United States. They may have a common ancestor, and *Protoparvovirus* may have undergone cross-species transmission. ParvoviridaeDogfe368C2 is 99.75% identical to the parvovirus of a bird (GenBank no. MV046412) collected in China. They may have a common ancestor, and unclassified *Parvoviridae* may have undergone cross-species transmission. The classification standard of viruses belonging to the *Parvoviridae* family is that members of the same genus should have at least 35%–40% amino acid sequence identity, and based on the coverage of NS1 protein >80%, if the NS1 protein of parvoviruses has more than 85% amino acid sequence identity, it can be considered as members of the same species (63). ParvoviridaeDogfe322C1 and ParvoviridaeDogfe362C9 in *Bocaparvovirus* form two new branches in the phylogenetic tree with identities of less than 60%, which are assumed to be novel species. ParvoviridaeDogfe340C1 in *Bocaparvovirus* forms one new branch in the phylogenetic tree with identities of less than 55%, which is assumed to be a novel species. The 11 parvoviruses clustered in *Dependoparvovirus* (e.g., ParvoviridaeDogfe346C1, ParvoviridaeDogfe385C1, ParvoviridaeDogfe383C2) formed a new cluster branch in the tree, and all share less than 70% identity, presuming that they compose a new genus.

### Canine CRESS-DNA viruses

**Fig 10:**
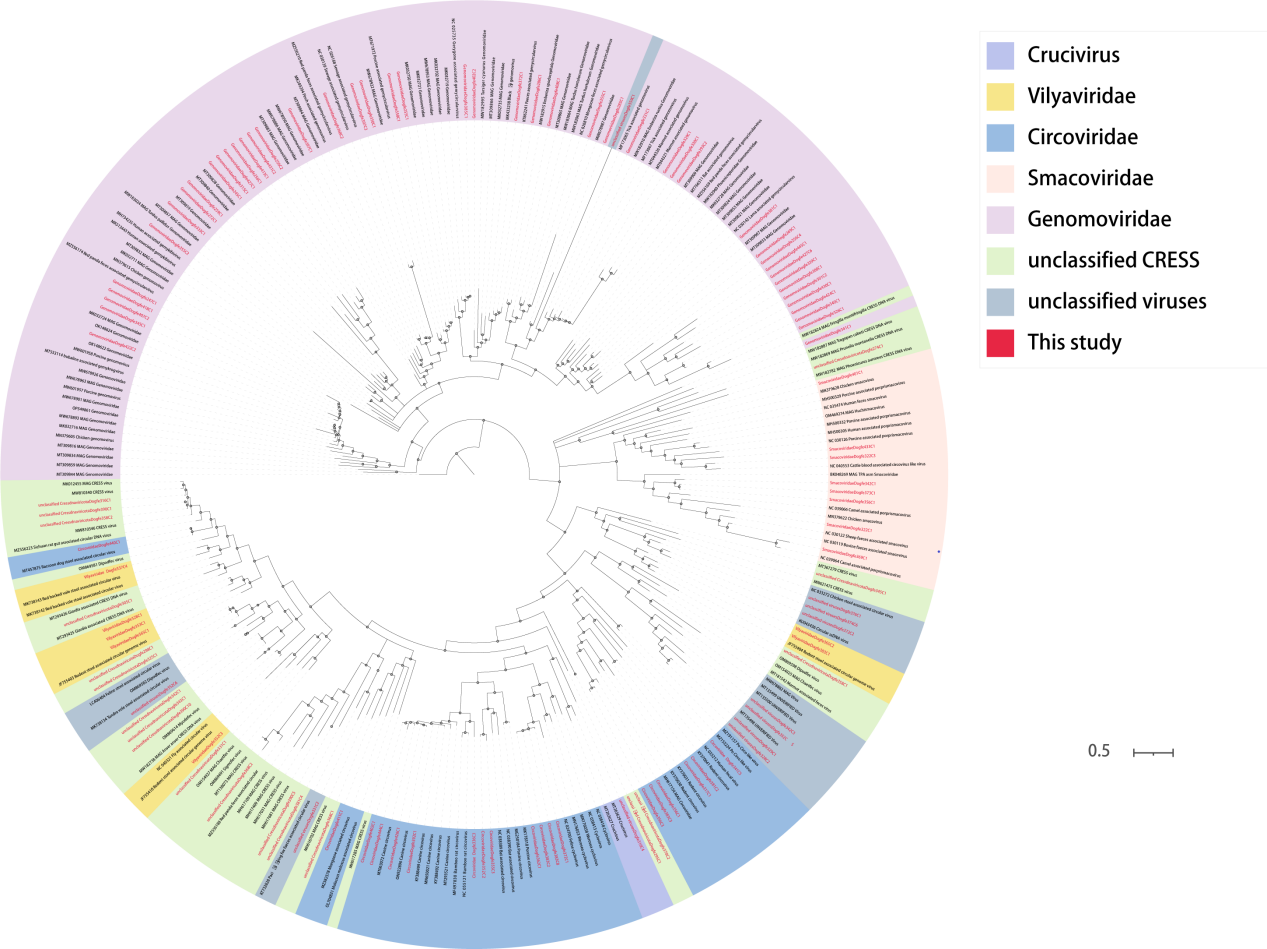
Phylogenetic relationship of CRESS-DNA viruses. Phylogenetic analysis trees were constructed based on the amino acid sequences of Rep proteins for circoviruses, picornaviruses, and other CRESS-DNA viruses. Red represents this study. The scale bar represents the length of the unit representing the value of the difference between organisms or sequences, equivalent to the scale of an evolutionary tree.

In recent years, with the development of viral metagenomics, an increasing number of unknown viruses have been identified, especially with a large number of unknown CRESS-DNA viruses in the animal digestive tract, and the ICTV continues to increase for the classification of CRESS-DNA viruses to meet the constantly newly discovered CRESS-DNA viruses (64). This study investigated CRESS-DNA viruses (including the *Smacoviridae*, *Vilyaviridae*, *Circoviridae*, *Genomoviridae*, and unclassified CRESS-DNA viruses) and unclassified viruses. We performed analysis on collected canine fecal samples, which yielded *Circoviridae* (*n* = 19), *Genomoviridae* (*n* = 46), *Smacoviridae* (*n* = 8), unclassified CRESS-DNA viruses (*n* = 19), *Vilyaviridae* (*n* = 7), and unclassified viruses (*n* = 11). For these viruses, a phylogenetic tree was constructed based on their rep proteins. The 46 viral genome genes belonging to the *Genomoviridae*, some clustered together and some individually with known viruses, form a total of 20 clades (Fig. 10). The eight viral genomes that are classified under the *Smacoviridae* family have clustered together into five distinct clades. The species and genus standard demarcations for *Smacoviridae* were based on the whole genome and rep amino acid sequences with cutoffs of 77.0% and 40.0%, respectively (65). According to the above indicators, no new genus was found among these eight viruses. The 19 viral genomes, which belong to the family *Circoviridae*, have been found to segregate into seven distinct clades. CircoviridaeDogfe341C5 only had 39.09% identity, which can assume an unmet species. A large number of CRESS-DNA viruses were obtained in this study, but we found that when phylogenetic trees were constructed between these viruses and the known CRESS-DNA viruses, the boundaries of the partial CRESS-DNA viruses in terms of virus classification were not very clear. A number of unclassified CRESS-DNA viruses and unclassified viruses were also identified in this study, contributing to the later classification of unclassified viruses with the possibility of new families or genera or species.

## DISCUSSION

Emerging infectious diseases are becoming more prevalent for various reasons, such as the growing trend of globalization, climate change, and other factors. Zoonoses, which are diseases that can be transmitted from animals to humans, make up the majority of these emerging diseases. These diseases are the result of intricate interactions between animals and humans involving pathogens (66). Since their domestication, dogs have been valued as companion animals, providing humans with emotional support and assistance. The relationship between humans, companion animals, and the natural environment they share is complex, and recent studies have shown that viruses from companion animals can cause zoonotic diseases or transmit other diseases to livestock, leading to serious disruptions in human daily life and impacting physical and mental health (67). Rabies is a well-known zoonosis, with patient case fatality rates approaching 100% and approximately 59,000 deaths annually worldwide (68). The virus is not limited to circulation among dogs alone but is also present among wild animals such as foxes and coyotes. To prevent the virus from spreading to other wild animals and triggering viral variants that may increase the risk of virus transmission to humans, measures must be taken. For instance, bat variant rabies caused a significant number of rabies cases between 1950 and 2007 (69). Although the natural reservoir of influenza A virus is not dogs, it is currently spillover to dogs due to the strong mutational capacity of viral genes (70). So research on the various viruses carried by dogs is imminent. A comprehensive understanding of all microbial communities of the canine digestive tract is possible through viral metagenomic techniques, helping us to study the genetic structure of known or unknown viruses (22).

To explore the viral composition present in the digestive tract of canines and to uncover potential viral threats to human health, the study utilized a total of 1,970 samples, comprising 1,020 canine fecal samples obtained from Guoluo and 950 from Yushu. The findings of the study were significant as they revealed the existence of 203 viral genomes.

Hepatitis E viruses infect a variety of animals but have not yet been identified in dogs (48), and the new hepatitis E virus identified in this study demonstrates the first hepatitis E virus discovery in dogs. The transmission route of HEV consists of many, mainly through drinking contaminated water sources and eating raw or undercooked animals (71). As canines are carriers of HEV, their excretions can contaminate rivers and other water sources, posing a threat to the health of both humans and domesticated animals. Therefore, it holds immense importance to detect the presence of HEV in dogs, particularly in the plateau region of China, where living conditions and habits facilitate the transmission of HEV. Furthermore, Yushu and Guoluo are the main birthplaces of the Yangtze River Basin and Yellow River Basin in China (72, 73), which, if contaminated, would trigger drinking water problems for a large number of residents downstream, causing serious social harm.

Our study led to the discovery of numerous novel astroviruses, and we were able to establish a plausible evolutionary linkage between the newly identified astroviruses and those found in different mammalian species, based on a thorough phylogenetic analysis. Furthermore, by employing taxonomic mapping from the ICTV, we were able to define a new genus and species that are likely to emerge (40). Through phylogenetic analysis, we found that certain newly discovered astroviruses exhibit a close evolutionary relationship with human astroviruses. Considering that they may have the ability to transmit to human beings, further research should be carried out on the higher-order structure and function of their proteins in subsequent studies to investigate whether they really have the potential to infect human beings.

Our study revealed a significant number of newly discovered viruses that exhibit a high degree of identity with known viruses. Notably, a novel coronavirus of canine origin was identified, sharing 92.29% identity with a murine coronavirus (GenBank no. MT820627), demonstrating that the two share a common evolutionary ancestor. It demonstrated that different species can be transmitted after coronavirus variation, which has implications for studying cross-species transmission of coronaviruses. Furthermore, the 11 anelloviruses found in the study were all more than 99% similar to anellovirus of murine (GenBank no. UVD40854), considered likely to have the capacity for cross-species transmission, and demonstrated a high degree of variability in the anelloviruses. Both Picornaviridaedogfe334C1 and Picornaviridaedogfe338C7 are more than 98% identical to picornaviruses of bovines. Additionally, Picornaviridaedogfe449C1 was 99.11% identical to picornavirus of canine (GenBank no. JN819204) collected in Hong Kong, China, in 2008. The high degree of identity of these viral sequences provides evidence that picornaviruses are widespread worldwide, from plateaus to plains, with the capacity for cross-regional and cross-species transmission. The discovery of these viruses helps us to study the origin of virus evolution and the direction of future evolution while providing evidence for virus cross-species transmission.

A total of 45 parvoviruses belonging to eight genera were identified in this study, of which viruses from three genera*, Bocaparvovirus* (*n* = 7), *Dependoparvovirus* (*n* = 17), *Protoparvovirus* (*n* = 8), can infect humans (62). These 32 canine *Parvovirinae* show homology between mammalian and avian sequences at the amino acid level. Based on classification rules for *Parvovirinae*, we hypothesized three new species and one new genus. Moreover, greater than 99% identity with known viruses was also found in the newly identified *Parvovirinae*, which may demonstrate cross-species transmission of *Parvovirinae*. The present study also identified a number of CRESS-DNA viruses and unclassified viruses, contributing to the later classification of unclassified viruses with the potential for the emergence of new families or genera or species.

In conclusion, this preliminary study of viruses isolated from the digestive tract of dogs in the plateau area identified 203 novel viral genomes in 1,970 samples. These viruses are divided into 11 known families and 2 unclassified groups. The genus and species of the new viruses were found in the digestive tract of these dogs, while the possibility of cross-species transmission of viruses was demonstrated. Our study enriched the viral community in the digestive tract microbiome of dogs and found types of viruses that threaten human health, providing technical support for the prevention and control of early warning of diseases caused by environmental contaminant viruses. However, we do not know much about the higher-order structure, physiological role, and pathogenic mechanism of the proteins of these viruses, and further experiments are needed to explore.

## Data Availability

The viral metagenomic data used to support the findings of this study have been deposited in the National Center for Biotechnology Information. The Sequence Read Archive (SRA) has received quality-filtered sequence reads that are listed under the BioProject ID PRJNA908720 and the BioSample ID SAMN32027925. All novel genes were accepted by the National Center for Biotechnology Information. The serial numbers were OQ198019–OQ198039, OQ198041–OQ198053, OQ198055–OQ198084, OQ198086–OQ198190, OQ198192–OQ198195, OQ198197–OQ198199, OQ198202, OQ198204–OQ198213, OQ198215, OQ198216, OQ198218–OQ198221, OQ198223–OQ198231, and OQ686762. There were no access restrictions.
